# The Moral Call to Action: A Special Issue of *HLRP* in Honor of Dr. Rima Rudd

**DOI:** 10.3928/24748307-20240620-01

**Published:** 2024-07

**Authors:** Maricel G. Santos, Michael Paasche-Orlow

With this Special Issue of *HLRP: Health Literacy Research and Practice*, we honor Dr. Rima Rudd, a dedicated scholar-practitioner whose work has been pivotal in forging health literacy as a field of study and practice. Dr. Rudd has retired after more than 30 years of service at Harvard University. As Dr. Rudd is one of the founding members of *HLRP'*s editorial board, the idea of a special tribute was an obvious one. This issue also looks beyond her association with *HRLP* and celebrates her lifelong efforts to elevate the field of health literacy studies. Her work in the United States and around the world led to the creation of new policies, calls to action, research agendas, assessment tools, and professional development initiatives in schools of public health, medicine, and dentistry, and more. Indeed, the celebration of Dr. Rudd's retirement provides us with a rare opportunity to take the long view on health literacy research and innovation: What lessons can we learn about the need for empowering adult learners and the substantive effort to remove health literacy barriers across health and public health settings from her scholarly trajectory?

Our readers will find answers to this question in this Special Issue. We hope this collection offers important lessons about the past and the future for all of us, including those readers who may be relatively new to health literacy and may not have had the opportunity to work with Dr. Rudd. This collection invites reflection on the scope of her leadership and should also spark questions of where we are (and should be) heading as a field. For many years, health providers have thought about health literacy as something that can be mitigated by improving our patient education materials and with clear communication by clinicians, who may even confirm comprehension. In such ways and others, we aim to remove barriers caused by low personal and organizational health literacy. Dr. Rudd aspired for much more than this. She anticipated that a focus on reforming materials and communication scripts would be valuable but would only have limited impact. In their tribute essay “Health Literacy for Healthy People: The Legacy of Dr. Rima Rudd,” Howard K. Koh, Cindy Brach, Emmeline Ochiai, Jennifer A. Bishop, and Carter Blakey describe why Dr. Rudd is the OG of the concept “organizational health literacy.” [Despite our field's penchant for eschewing jargon and initialisms, OG is purposefully included here to make Dr. Rudd laugh with happiness]. Koh and colleagues also applaud Dr. Rudd for exposing “the chasm between people's capacity to process critical health information and the health profession's ability to deliver such information effectively, empathetically, and equitably and then [working] over a lifetime to narrow that chasm.” We certainly hope *HLRP* readers will hear a call to action in the words of Howard K. Koh and colleagues, and “continue to ‘expose chasms’ in health literacy in contexts that are not already well-represented in the *HLRP*, and bring greater visibility to the communities we seldom hear from.”

New thinking about ‘chasms’ is evident in the article, “Racial Composition of Past and Current Social Environments and Health Literacy,” by Jemar R. Bather, Feng Liu, Melody S. Goodman, and Kimberly A. Kaphingst, which examines the relationship between the racial composition of one's social environments, past and present, and health literacy outcomes. Long term, and even intergenerational effects, have been seen in health literacy. As we work to find solutions in health and public health, we cannot lose sight of underlying inequities in other sectors, such as housing, education, and the carceral system that also perpetuate racial and ethnic health disparities. Intersectoral interventions will be needed. Readers will find a wonderful example of intersectoral potential in the article “Rudd's Organizational Health Literacy Scholarship: The Maryland Experience,” by Cynthia Baur, Catherine Maybury, Lindsay Rosenfeld, and Leah Richey; it is a chronicle of important advancements in assessment, health care delivery, and organizational capacity that reflect Dr. Rudd's leadership and collaborative spirit.

This Special Issue also recognizes Dr. Rudd for her efforts to press for robustness and integrity in health literacy research and research-to-practice translations, as captured in a Mission and Motivation article by Kristine Sørensen and a Short Reflection Essay by Terri Ann Parnell. When you read these essays, you will hear the admiration of her students and colleagues. We hope you will also be called to bring rigorous research methods to bear and to expand the contexts where a focus on health literacy can promote health equity. This issue highlights work carried out in the U.S., Brazil, and Japan, reflective of the broad reach of Dr. Rudd's influence.

A dedicated Freirean (Paulo Freire) scholar, Dr. Rudd understood that both community engagement and policy change are integral to health promotion and a more just health care system (Rudd & Comings, 1994). It is fitting that this Special Issue includes two articles that draw attention to her commitment to Freirean ideals, namely community empowerment and patient empowerment. Darriel B. Harris and Debra L. Roter pay tribute to Dr. Rudd's Freirean ideals in their Brief Report “Profound Love and Dialogue: Paulo Freire and Liberation Education,” which provides a fresh commentary on the continued relevance of Freire in contemporary health literacy scholarship and practice. Also, not to be missed is the authors' answer to the question, “What does love have to do with it?”, essential reading for anyone not familiar with Freire's work.

Both Dr. Rudd and Debra L. Roter brought the philosophy and practice of Paulo Freire into the room whenever they could, frequently by name—and by daring to teach. They showed generations of students how investment in the adult education system and health literacy interventions are forms of empowerment and critical public health endeavors (see Danielle Marie Muscat's Short Reflection Essay “Harnessing the Qualities and Principles of Adult Education for Health Literacy Learning” in this Special Issue). Dr. Rudd in collaboration with many colleagues provided the field of health literacy with a kind of uplifting optimism that attracted people who wanted to improve the world. We are going to need to find new ways to help the field grow now that these two health literacy influencers have retired.

Also inspired by Freirean pedagogy is the article, “Qualitative Evaluation of a Health Literacy Program for Older Adults in a Community Dwelling in Brazil,” by Andreivna Kharenine Serbim, Julie Ayre, Lisiane Manganelli Girardi Paskulin, Don Nutbeam, and Danielle Marie Muscat. This study describes the impact of a health literacy program on a low-resourced community of older adults affiliated with a primary care health unit in Arapiraca, Brazil. The authors demonstrate the social dimensions of improved health literacy outcomes, including the role of social cohesion, social support, and “co-learning” dynamics (the capacity for community members to facilitate one another's health literacy learning). These findings hold echoes of Dr. Rudd's words in 2002 about the conditions that accelerate the diffusion of innovation:

The underlying theoretical assumption [in the diffusion of innovation model] is that change is promoted through ideas or information introduced by people with whom you can identify….This model is very useful in helping us answer two critical questions: How do ideas spread among a group of people over time? How can we speed up this process?

(Rudd, 2002, p. 8).

Without a doubt, Dr. Rudd has created her own vectors of diffusion. She mentored and supervised generations of graduate students, health practitioners, and clinicians and shaped their capacity as change agents in health care. This impact is captured in “Six-Word Memoirs of a Mentor,” by Lindsay Rosenfeld and Vanessa Watts Simonds and a Short Reflection Essay, “Rigor, Dignity, and Collaboration: A Tribute to Dr. Rima Rudd,” by Lindsay Rosenfeld and Catherine Leslie. Cynthia Baur reminds us in her tribute Short Reflection Essay, “Reflections on Dr. Rima Rudd's Significance to Health Literacy in the Shadow of Health Equity,” that Dr. Rudd's career must be viewed as a celebration of women in leadership positions, both in higher education and public health policy: “We forget how much women now in their 70s, 80s, and 90s have had to overcome to create rich professional lives... We should pause in respect for what it took for Dr. Rudd, her peers, and those before her to forge a life of the mind, to be working professionals and to build the careers they now step away from. It wasn't easy for them.”

Dr. Rudd continues to be a fierce champion of clarity—clarity in communication, clarity in purpose. And while many health communication experts discourage the use of acronyms for their potential to hinder communication, we feel there is one acronym that serves our purposes well:

**R**eal, **I**nsight, **M**otivates, **A**ction, **R**ealistic, **U**nderstanding, **D**etermines, **D**irection.

Congratulations, Dr. Rima Rudd. We extend our deep appreciation to everyone who contributed to this Special Issue, and in helping us to honor your legacy.
